# Single-Leg Weight-Bearing Reveals Hidden Varus Deformity in Knee Osteoarthritis

**DOI:** 10.3390/jcm15218284

**Published:** 2026-10-24

**Authors:** Cihangir Turemis, Firat Erpala, Tahir Ozturk, Orhun Ufuk Tipi, Mehmet Erduran, Ibrahim Halit Pinar

**Affiliations:** 1 Department of Orthopaedics and Traumatology, Cesme Alper Cizgenakat State Hospital, 35930 Izmir, Turkey; cihangirturemis@gmail.com; 2 Department of Orthopaedics and Traumatology, Memorial Hospital, 34180 Istanbul, Turkey; ozturk.tahir@yahoo.com; 3 Department of Orthopaedics and Traumatology, Dokuz Eylul University, 35340 Izmir, Turkey; ufuk.tipi@gmail.com (O.U.T.); dr.erduran@gmail.com (M.E.); cihangir_19@hotmail.com (I.H.P.)

**Keywords:** knee osteoarthritis, varus malalignment, single-leg stance, mechanical medial proximal tibial angle, joint line convergence angle, biomechanics

## Abstract

**Background/Objectives**: Accurate radiographic assessment of coronal alignment is essential for corrective osteotomies and knee arthroplasty. However, conventional bilateral weight-bearing radiographs may underestimate varus alignment under single-leg weight-bearing. This study aimed to describe the increase in knee varus alignment between bilateral and single-leg weight-bearing stances and to evaluate the factors associated with this increase. **Methods**: Standardized long-leg radiographs were obtained in bilateral and single-leg weight-bearing stances using a digital radiography system. ΔVarus was defined as the difference between single-leg bearing and bipedal varus angles (single-leg bearing varus—bipedal varus) and was reported in degrees (°). Patients were stratified by dominant lower extremity and sex for subgroup analyses. **Results**: The final sample study groups consisted of 101 patients (57 women, 44 men) with a mean age of 57.0 ± 12.7 years and a mean BMI of 25.1 ± 2.7 kg/m^2^. Analysis of the entire cohort revealed a statistically significant increase in varus alignment following single-leg weight-bearing (*p* < 0.001). Right dominant individuals had a significantly greater varus angle in the right lower extremity (10.9 ± 1.9°) than in the contralateral limb (9.9 ± 2.3°; *p* = 0.016). Similarly, in left-dominant individuals, the varus angle of the left knee (10.9 ± 2.3°) was significantly greater than that of the right knee (9.4 ± 1.8°; *p* = 0.009). The mean ΔVarus was 1.54° (SD, 0.75°; 95% CI, 1.39–1.69°; *p* < 0.001) for the right knee and 1.30° (SD, 0.70°; 95% CI, 1.16–1.44°; *p* < 0.001) for the left knee. **Conclusions**: This study has shown that standard bilateral weight-bearing long-leg radiographs may not fully reflect knee varus deformity, particularly the functional coronal alignment changes that occur during single-leg weight-bearing. An additional varus deformity of approximately 1.6° was observed in the dominant limb during single-leg weight-bearing. Single-leg bearing radiographs demonstrated greater varus alignment than bipedal radiographs. However, the clinical implications of this radiographic difference for surgical planning remain undetermined. In future prospective studies, the combined evaluation of gait analysis and single-leg loading radiographs will better elucidate the true contribution of the dominant limb to functional knee biomechanics.

## 1. Introduction

Accurate assessment of mechanical impairment and coronal alignment of the lower extremity is a cornerstone of success in reconstructive knee surgery. Whether high tibial osteotomy (HTO), unicompartmental knee arthroplasty or total knee arthroplasty (TKA) is planned, the surgeon must accurately determine the contributions of bone anatomy and soft tissue structure to the overall varus or valgus deformity [1,2,3]. Underestimating the magnitude of misalignment or failing to recognize dynamic multiplane instability can lead to failures resulting in suboptimal implant placement, unstable soft tissue sheaths, accelerated poly-wear, and early catastrophic outcomes [4,5]. Current standard care features full-length, hip-to-ankle long-leg radiographs obtained during static, bilateral leg weight-bearing [6]. This projection provides a basic structural map while distributing body weight equally between both limbs. However, bilateral standing radiographs represent a static loading condition and may not fully reflect the alignment observed when the knee is subjected to unilateral loading. Consequently, the complex dynamic forces generated during the phase of walking on one leg may not be fully reproduced by bilateral weight-bearing radiographs; during this phase, the body continues to be regularly subjected to vertical loads that it can withstand three to four times its weight [7]. During one-legged loading, a significant medial shift in the ground reaction force vector creates a dynamic varus adduction moment. In knee osteoarthritis (OA), this mechanism may accentuate medial compartment loading and is clinically expressed during gait as a dynamic varus thrust [2,8].

The magnitude of coronal alignment observed on weight-bearing radiographs may reflect both osseous geometry and the contribution of intra-articular and soft-tissue structures [9]. It can be theorized that the geometric shape of this varus deformity is determined by specific local influences: proximal morphology, represented by the mechanical medial proximal tibial angle (mMPTA), distal femur morphology, represented by the mechanical lateral distal femoral angle (mLDFA), joint line convergence, represented by the joint line convergence angle (JLCA), and sagittal represented by the posterior tibial slope (PTS) [10,11,12]. In particular, the JLCA may reflect the contribution of intra-articular deformity and soft-tissue laxity to the overall coronal alignment, whereas mMPTA and mLDFA primarily describe the osseous components of alignment [10,11,12]. Thus, changes in alignment under different weight-bearing conditions may arise from the interaction of underlying bone geometry and load-dependent joint behavior.

Importantly, static radiographic varus alignment should be distinguished from dynamic varus thrust. Varus thrust describes a dynamic lateral movement of the knee during the stance phase of gait and reflects the interaction between limb alignment, joint loading, and soft-tissue and neuromuscular factors [7,8]. In contrast, a single-leg weight-bearing radiograph represents a static assessment obtained under unilateral loading and should not be considered a direct radiographic measurement of varus thrust. Nevertheless, it may provide information regarding changes in coronal alignment when the loading condition is altered.

Although the effects of loading conditions on knee biomechanics and coronal alignment have been investigated, direct comparisons of alignment measurements obtained from bilateral and single-leg weight-bearing long-leg radiographs remain limited. Similarly, the extent to which the change in varus alignment under unilateral loading is associated with limb dominance and specific osseous or radiographic parameters has not been clearly established. This represents an important knowledge gap, particularly because differences between bilateral and single-leg measurements may influence the interpretation of lower-extremity alignment rather than simply reflecting a difference in underlying bony deformity.

The aim of this study was to compare knee varus alignment measurements obtained from standard bilateral weight-bearing and single-leg weight-bearing radiographs and to determine the magnitude of the change in varus alignment between these two loading conditions. The primary outcome was ΔVarus, defined as the single-leg weight-bearing varus angle minus the bilateral weight-bearing varus angle. Secondary analyses evaluated the association of ΔVarus with limb dominance and demographic and radiographic parameters, including age, body mass index (BMI), mMPTA, mLDFA, JLCA, PTS, and mechanical axis deviation (MAD).

## 2. Materials and Methods

### 2.1. Study Design and Participants

This single-center, cross-sectional observational study included patients who presented to our outpatient clinic between January 2022 and November 2025 and met the predefined inclusion criteria. Patients with symptomatic medial compartment knee osteoarthritis who met the inclusion criteria and had radiographic evidence of osteoarthritis in both knees were identified during their outpatient visits. After providing written informed consent, the patients underwent standardized bilateral and single-leg weight-bearing radiographic examinations. Their clinical and radiographic data were subsequently retrieved from the institutional records and retrospectively analyzed for the purposes of the present study. Formal ethical approval was obtained from the Institutional Review Board and Ethics Committee (Protocol No. 2025/14). All procedures were performed in accordance with the 1964 Declaration of Helsinki and its subsequent revisions.

Inclusion criteria were as follows: patients 18 years of age and older with symptomatic medial compartment knee OA (Kellgren-Lawrence grades II to IV in both knees); clinically or radiographically visible varus alignment in the lower extremity, availability of high-quality full-length lower extremity radiographs obtained under both bilateral and single-leg weight-bearing conditions; and willingness to participate in the study. Patients were excluded if they had a history of periarticular fracture, high-energy ligamentous knee injury, previous lower-extremity osteotomy or arthroplasty, inflammatory joint disease, active neuromuscular disease precluding independent standing, or inadequate-quality radiographs that prevented reliable measurement.

### 2.2. Radiographic Acquisition

Longitudinal radiographs (orthoradiographs) of both lower limbs were taken for patients with varus deformity attending the outpatient clinic. Standardized long-leg radiographs were obtained using a digital radiography system with a single film focal length. Patients were positioned with the feet directed forward and the lower extremities in a standardized position to minimize rotational variation.

**Figure 1:**
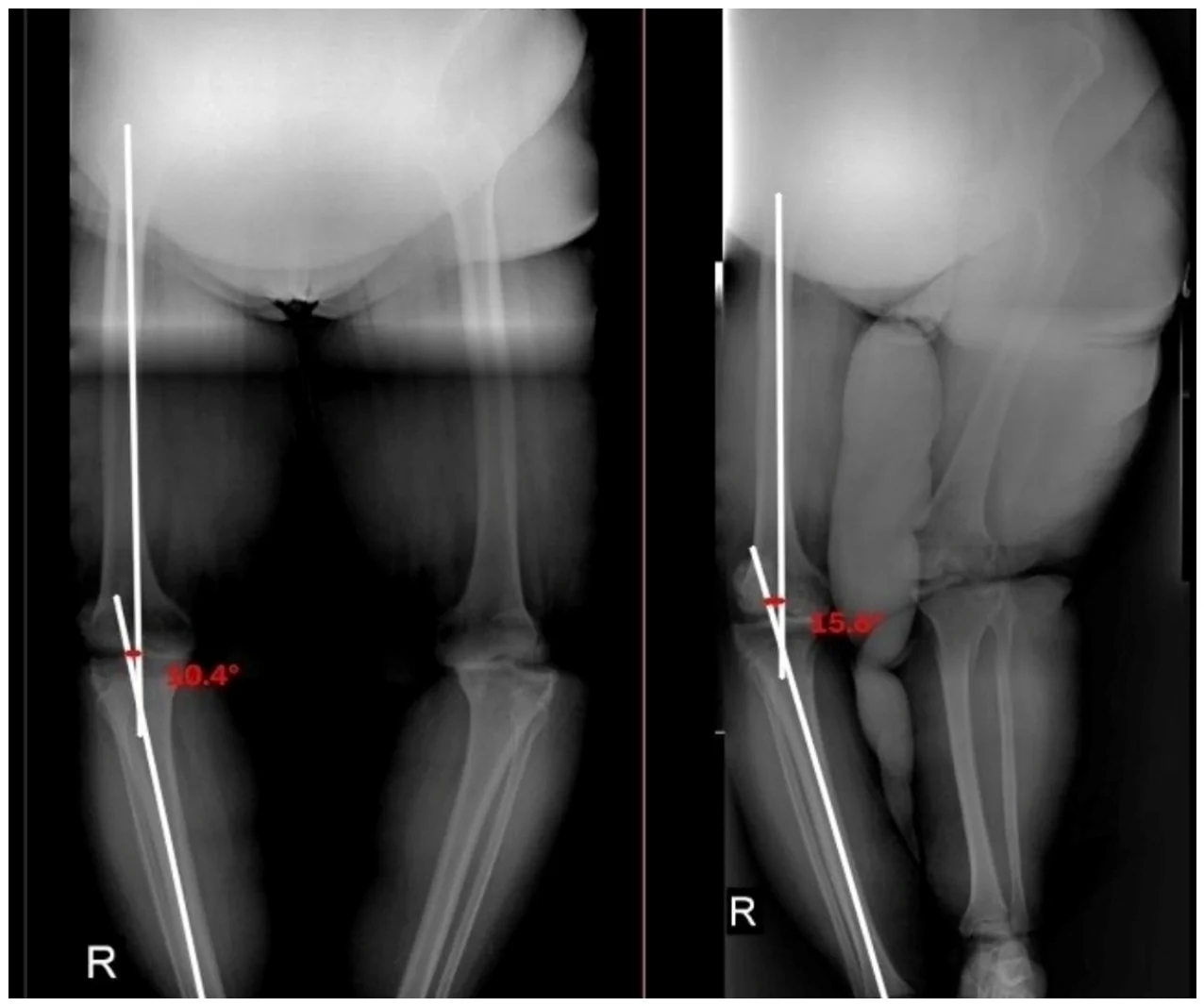
ΔVarus represents the change in varus alignment during single-leg weight-bearing compared with bilateral weight-bearing radiographs.

For the bilateral weight-bearing (bipedal stance) view, patients stood upright with both knees fully extended and their body weight distributed as evenly as possible between both lower extremities. For the single-leg weight-bearing view, patients were instructed to transfer their full body weight onto the target limb while maintaining the knee in full extension, with the contralateral limb lifted from the floor. No external support was permitted during image acquisition, and patients were required to maintain the single-leg stance independently. Standard lateral knee radiographs were obtained in a true lateral projection with the knee flexed to approximately 30°. The change in varus angle (ΔVarus) following weight-bearing was calculated and used for subsequent analyses (Figure 1).

### 2.3. Radiographic Measurements

**Figure 2:**
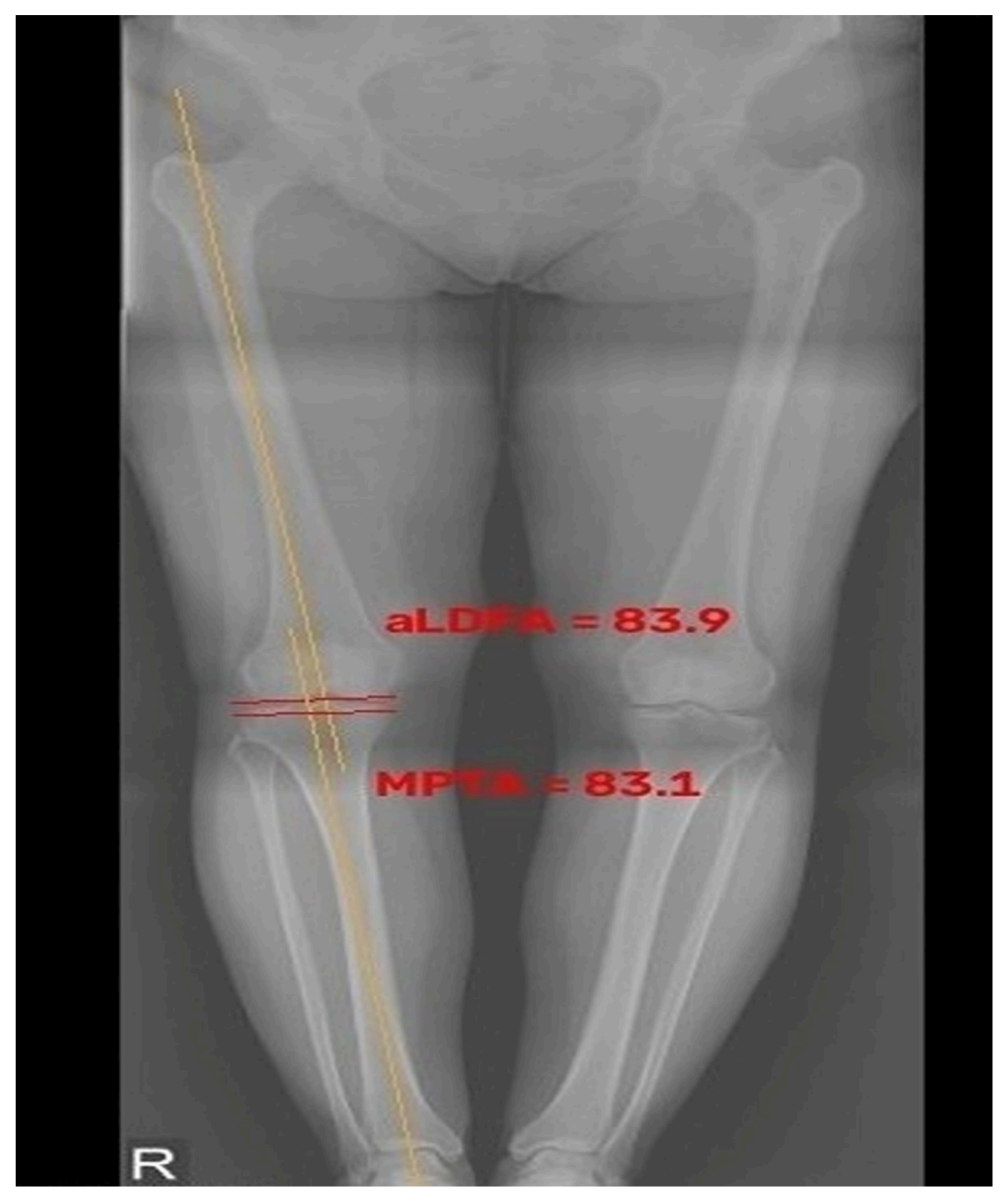
Anteroposterior standing long-leg radiograph showing mLDFA and mMPTA measurements.

The primary radiographic outcome was the coronal-plane varus angle measured on full-length standing anteroposterior radiographs using the hip–knee–ankle (HKA) mechanical axes. The femoral mechanical axis was defined as the line connecting the center of the femoral head to the center of the knee, whereas the tibial mechanical axis was defined as the line connecting the center of the knee to the center of the ankle. (Figure 2) Varus alignment was expressed in degrees (°), with higher values indicating greater varus alignment.

The change in varus alignment (ΔVarus) was calculated as the difference between the single-leg and bilateral weight-bearing varus angles:

ΔVarus (°) = Single-leg weight-bearing varus angle (°) − Bilateral weight-bearing varus angle (°).

A positive ΔVarus value indicated an increase in varus alignment under single-leg weight-bearing.

The mechanical medial proximal tibial angle (mMPTA) was defined as the medial angle between the tibial mechanical axis and the proximal tibial joint line. The mechanical lateral distal femoral angle (mLDFA) was defined as the lateral angle between the femoral mechanical axis and the distal femoral joint line. Both parameters were measured separately for the right and left knees on full-length anteroposterior radiographs obtained under both bilateral and single-leg weight-bearing conditions and expressed in degrees (°).

The joint line convergence angle (JLCA) was defined as the angle between the distal femoral and proximal tibial articular surfaces in the coronal plane and expressed in degrees (°). Mechanical axis deviation (MAD) was defined as the perpendicular distance between the center of the knee and the lower-limb mechanical axis connecting the center of the femoral head to the center of the ankle. MAD was expressed in millimeters (mm). The posterior tibial slope (PTS) was measured on lateral knee radiographs obtained with the knee flexed to approximately 30°. It was defined as the angle between the tibial plateau and the tibial reference axis in the sagittal plane and expressed in degrees (°).

JLCA and MAD were measured once on full-length anteroposterior radiographs, whereas PTS was measured once on a lateral knee radiograph. These parameters were not reassessed separately under bilateral and single-leg weight-bearing conditions. Each was recorded as a single patient-level radiographic variable and used as a common variable in the correlation analyses of both right and left ΔVarus.

Limb dominance was determined by asking patients which lower extremity they preferentially used to kick a ball or another object or to step onto a vehicle. Patients were subsequently classified as right- or left-limb dominant. Analyses were performed according to limb dominance and sex.

### 2.4. Radiographic Assessment and Blinding

All radiographic measurements were performed by a musculoskeletal radiologist (AB) with 22 years of experience. To minimize observer bias, the radiologist assessed the radiographs using anonymized study images in which the patients’ clinical records and demographic information were unavailable. The images were presented without information indicating the weight-bearing condition. Each radiographic parameter was measured once by the same radiologist; therefore, intra-observer reliability was not assessed.

### 2.5. Statistical Analysis

The data were analyzed using SPSS Statistics Software (version 23.0, IBM Corp., Armonk, NY, USA). The distribution of the data was evaluated with the Kolmogorov–Smirnov test. Continuous variables were expressed as mean ± standard deviation or median (minimum–maximum), according to data distribution. Categorical variables were presented as frequencies (*n*) and percentages (%). The categorical data were assessed with the Pearson chi-square test. The parametric and non-parametric data were evaluated with Student’s *t*-test and Mann–Whitney U test, respectively. Because the bilateral and single-leg radiographs were obtained from the same knees and patients, comparisons between bilateral and single-leg weight-bearing measurements were treated as paired observations and analyzed using the Wilcoxon signed-rank test. Relationships between continuous variables were assessed using Spearman’s rank correlation analysis. Spearman’s rank correlation coefficients were used to assess associations between radiographic parameters and ΔVarus. Two-tailed *p* values were obtained from the standard Spearman correlation test, while 95% confidence intervals for correlation coefficients were estimated using 1000 bootstrap samples. Multivariable linear regression analyses were performed separately for right and left ΔVarus as the dependent variables. Age, BMI, mMPTA, mLDFA, JLCA, MAD, and PTS were included as independent variables. A *p* value of < 0.05 was considered statistically significant for all analyses.

## 3. Results

The final study sample consisted of 101 patients (57 women, 44 men) with a mean age of 57.0 ± 12.7 years and a mean BMI of 25.1 ± 2.7 kg/m^2^. Of the patients, 45.5% (*n* = 46) had a normal BMI, whereas 54.5% (*n* = 55) were classified as overweight or obese (BMI ≥ 25 kg/m^2^). Correlation analysis demonstrated no statistically significant association between BMI and right (*p* = 0.553) or left (*p* = 0.637) knee single-leg weight bearing varus angle, right (*p* = 0.299) or left (*p* = 0.900) ΔVarus, right (*p* = 0.093) or left (*p* = 0.170) mMPTA, right (*p* = 0.093) or left (*p* = 0.958) mLDFA, JLCA (*p* = 0.937), MAD (*p* = 0.493), or PTS (*p* = 0.249) measurements (all *p* > 0.05).

Right-limb dominance was observed in 74.3% (*n* = 75) and left-limb dominance in 25.7% (*n* = 26) of the population. The mean varus angle increased from 9.2 ± 1.8° under bilateral weight-bearing conditions to 10.6 ± 2.1° under single-leg weight-bearing conditions in the right knee, corresponding to a mean ΔVarus of 1.54 ± 0.75° (95% CI, 1.39–1.69°; *p* < 0.001). In the left knee, the mean varus angle increased from 8.5 ± 2.0° under bilateral weight-bearing conditions to 9.7 ± 2.1° under single-leg weight-bearing conditions, with a mean ΔVarus of 1.30 ± 0.70° (95% CI, 1.16–1.44°; *p* < 0.001). ΔVarus was calculated as the single-leg weight-bearing varus angle minus the bilateral weight-bearing varus angle, with positive values indicating an increase in varus alignment under single-leg weight bearing.

In the limb-dominance analysis, among right-limb-dominant patients (*n* = 75), the single-leg weight-bearing varus angle was significantly greater in the dominant right knee (10.9 ± 1.9°) than in the contralateral left knee (9.9 ± 2.3°; *p* = 0.016). Similarly, among left-limb-dominant patients (*n* = 26), the single-leg weight-bearing varus angle was significantly greater in the dominant left knee (10.9 ± 2.3°) than in the contralateral right knee (9.4 ± 1.8°; *p* = 0.009). In a paired comparison of the dominant and non-dominant limbs, ΔVarus was significantly greater in the dominant limb than in the non-dominant limb (1.65 ± 0.74° vs. 1.20 ± 0.66°; Wilcoxon signed-rank test, *p* < 0.001). The mean paired difference was 0.46° (95% CI, 0.27–0.64°).

When patients were grouped according to sex, no statistically significant differences were observed between the groups in terms of mean age, dominant lower extremity, or radiographic measurements (Table 1).

Analysis of the entire cohort revealed a statistically significant increase in varus alignment following single-leg weight-bearing (Table 2).

The correlations between ΔVarus of the right and left knees and the measured radiographic parameters are presented in Table 3. Among the evaluated parameters, posterior tibial slope (PTS) demonstrated a weak inverse correlation with ΔVarus (Spearman’s ρ = −0.199, *p* = 0.046). No statistically significant correlations were observed between ΔVarus and the other evaluated radiographic parameters.

Multivariable linear regression models were constructed using age, BMI, and radiographic parameters to identify independent predictors of right and left knee ΔVarus (Table 4). None of the evaluated variables demonstrated a statistically significant independent association with ΔVarus in either the right or left knee.

## 4. Discussion

The most significant finding of this study is that, compared with bilateral weight-bearing radiographs, the knee varus angle increases significantly during single-leg weight-bearing. When the entire cohort was evaluated, single-leg weight-bearing resulted in an additional increase of approximately 1.3–1.5° in varus deformity. Although the absolute magnitude of this change was relatively small, the finding was consistent in both knees and suggests that the loading condition may influence the radiographic assessment of coronal knee alignment. These results indicate that bilateral weight-bearing radiographs may underestimate the degree of varus alignment that becomes apparent under unilateral loading. As a significant proportion of daily activities involves periods of single-limb loading, single-leg weight-bearing may provide complementary information regarding load-dependent coronal alignment. However, whether this additional information improves surgical planning or changes treatment decisions remains uncertain and requires prospective evaluation. This finding may be relevant to preoperative alignment assessment in patients being considered for HTO or TKA; however, its implications for surgical templating and treatment decisions remain uncertain [1,3,6].

Lower limb alignment surgeries, particularly HTO and TKA, are not merely procedures based on bone resection, but reconstructive procedures aimed at the correct re-establishment of the mechanical axis and appropriate soft tissue balance [1,6,10]. For this reason, preoperative radiological assessment and templating are among the most critical stages of the surgery. Accurate assessment of the mechanical axis is an important component of preoperative planning for HTO and TKA [4,5]. However, although the additional varus observed during single-leg loading may be relevant when interpreting radiographic alignment, the present study did not determine whether this difference affects implant positioning, soft-tissue balancing, or postoperative outcomes. Standard planning methods currently in use are based on bilateral weight-bearing long-leg radiographs [3,6]. Our findings suggest that unilateral loading can reveal a modest additional varus displacement that is not fully represented under bilateral loading. This may be relevant in patients in whom the measured deformity is close to a surgical correction threshold, although the present study did not evaluate whether this difference alters osteotomy correction targets, arthroplasty planning, or postoperative outcomes. Therefore, the potential role of single-leg radiographs in HTO or TKA planning should be considered a subject for future investigation rather than a basis for routine clinical use.

Accurate assessment of lower limb alignment is important in the evaluation of medial compartment knee osteoarthritis and may contribute to preoperative planning for corrective osteotomy and knee arthroplasty. However, the clinical interpretation of alignment measurements should extend beyond group-level mean values and consider individual anatomical characteristics and potential alignment outliers. A recent systematic review and meta-analysis comparing robotic-assisted and conventional unicompartmental knee arthroplasty emphasized the importance of evaluating patient-specific anatomy and radiographic outliers rather than relying solely on mean postoperative alignment parameters [13]. In this context, the additional 1–2° of varus observed on single-leg weight-bearing radiographs in our study may be relevant when interpreting an individual patient’s coronal alignment. Nevertheless, the clinical significance of this difference remains uncertain, as our study did not evaluate changes in surgical planning, postoperative alignment, or clinical outcomes. Therefore, the observed differences should not be interpreted as evidence that surgical correction must be modified on the basis of single-leg radiographs alone. Further prospective studies are needed to determine whether these differences influence surgical decision-making and whether any resulting changes improve patient outcomes.

Recent studies have shown that in knee OA, the knee adduction moment and lower extremity loading during gait can show significant asymmetries between the two sides, and these biomechanical differences may be associated with disease progression [14,15]. However, almost all of these studies are based on gait analysis and movement laboratory data [2,7,16]. The biomechanical mechanisms described in these studies provide a possible context for our findings, but the present study should not be interpreted as a direct assessment of gait biomechanics. A standing single-leg radiograph represents a static snapshot of coronal alignment under unilateral loading and does not directly measure the knee adduction moment, gait asymmetry, or dynamic varus thrust. Especially in osteoarthritic knees, this loading pattern causes the development of dynamic “varus thrust” and may reveal load-dependent changes in coronal alignment that are not apparent under bilateral weight-bearing conditions [8,17]. Thus, our findings may represent a static radiographic correlate of load-dependent alignment changes, but they cannot establish that the observed increase in varus corresponds to dynamic varus thrust during walking. While Chang and Sharma’s long-term studies highlighted the importance of mechanical axis dysfunction in OA progression, Kuroyanagi et al. demonstrated that dynamic varus thrust is related to structural alignment parameters [2,8,17]. Future studies combining single-leg radiographs with three-dimensional gait analysis, force-plate measurements, or other biomechanical assessments are needed to determine whether the radiographic change observed in the present study corresponds to clinically relevant alterations in dynamic knee loading.

In our study, no significant association was found between body mass index and ΔVarus. It has previously been demonstrated in numerous studies that obesity is a significant risk factor for the development and progression of knee OA, and that it is associated with increased knee adduction moment and mechanical loading [2,7,16,17]. However, the majority of these studies have focused on gait analysis, disease progression or general mechanical loading. Our study, however, assessed the instantaneous radiographic coronal alignment changes that occur during single-leg weight bearing. The absence of a statistically significant association between BMI and ΔVarus in our cohort should therefore not be interpreted as evidence that body weight has no influence on functional alignment. The relatively narrow BMI distribution and limited representation of patients with severe obesity may have reduced our ability to detect such an association. The change in alignment observed during unilateral loading is likely to be influenced by multiple factors, including local bone geometry, ligamentous laxity, joint-line mechanics, and neuromuscular control, rather than body weight alone.

Another relevant finding was that ΔVarus was significantly greater in the dominant limb than in the non-dominant limb (1.65 ± 0.74° vs. 1.20 ± 0.66°, respectively; *p* < 0.001). Previous biomechanical studies have suggested that lower-limb dominance may influence joint kinetics, kinematics, and loading patterns during walking and single-limb tasks [18,19]. However, the present study did not directly assess gait biomechanics, proprioception, muscle strength, or neuromuscular control; therefore, the mechanisms underlying this difference remain uncertain. Paillard and Noé reported that the effects of limb dominance on single-leg balance performance may vary according to factors such as proprioception, muscle strength, and neuromuscular control [20]. In addition, coronal-plane loading and the knee adduction moment are important aspects of knee biomechanics in osteoarthritis [20,21]. Nevertheless, because these biomechanical factors were not directly measured in the present study, the greater ΔVarus observed in the dominant limb cannot be attributed to any specific mechanism. This finding should therefore be interpreted as a difference in load-related radiographic alignment rather than evidence of a confirmed functional or biomechanical advantage or disadvantage of the dominant limb.

Significant differences were not observed between female and male patients in terms of radiographic parameters and ΔVarus. Previous studies have suggested that functional deformity may be more closely related to local biomechanical characteristics and joint stability than to sex [2,16,17], no statistically significant differences in ΔVarus were detected between female and male patients in the present study. The absence of statistically significant sex differences in the present cohort should not be interpreted as evidence that sex has no influence on loading-related changes in coronal alignment. Rather, no significant association was detected within the characteristics and sample size of this study. Differences related to sex may become more apparent in larger cohorts incorporating measures of muscle strength, alignment, ligamentous laxity, or dynamic loading.

When the relationships between radiographic parameters and ΔVarus were examined, only a weak negative correlation was found with the posterior tibial slope (PTS). However, the low strength of this relationship and the fact that it lost its significance in multivariate analysis do not support the interpretation of PTS as an independent predictor of dynamic varus changes associated with single-leg weight-bearing. Accordingly, the observed association with PTS should be regarded as exploratory. Previous studies have shown that mMPTA and JLCA are associated with dynamic malalignment and functional coronal alignment [8,11,12,22]. JLCA is particularly relevant because it may reflect the soft-tissue component of coronal alignment and can change with loading. In the present study, however, JLCA was not significantly correlated with ΔVarus, suggesting that the additional varus observed during unilateral loading cannot be explained solely by changes represented by this parameter. However, in the majority of these studies, the endpoint assessed was absolute coronal alignment or dynamic malalignment, and the change in ΔVarus between bilateral and single-leg weight-bearing was not directly examined. This methodological difference may partly explain the inconsistency between our results and those of previous studies. Furthermore, the fact that our study included only a relatively homogeneous group of patients with medial compartment varus OA may have narrowed the distribution of structural parameters, thereby making it difficult to demonstrate their independent effects. Finally, the fact that no radiographic parameter was identified as an independent predictor of ΔVarus in the multivariate regression analysis suggests that the increase in functional varus observed during single-leg weight-bearing cannot be explained by static bone morphology alone; load-dependent soft-tissue behaviour and neuromuscular factors may also contribute to this response [8,11,12,22].

This study has certain limitations. Firstly selection bias cannot be entirely excluded because inclusion required the availability of both bilateral and single-leg weight-bearing radiographs; consequently, the study population may not represent all patients with medial compartment knee OA. Secondly, the relatively small number of patients reduces the generalisability of the results, and the findings need to be validated in a larger, multicentre series. Thirdly, radiographic measurements were performed by a single experienced musculoskeletal radiologist, and inter-observer and intra-observer reliability analyses could not be carried out. Consequently, measurement reproducibility could not be quantified, and it remains uncertain whether the observed angular differences of approximately 1–2° exceed potential measurement variability. Therefore, the magnitude of these differences should be interpreted cautiously. Furthermore, patients were not assessed in subgroups according to Kellgren-Lawrence staging; consequently, the effect of OA severity on dynamic varus changes associated with single-leg weight-bearing could not be analysed. In addition, clinical outcomes and surgical decision-making were not evaluated, and therefore the clinical significance of the observed radiographic difference remains uncertain. Finally, whilst single-leg weight-bearing radiographs reflect functional loading better than bilateral radiographs, they do not fully represent actual gait biomechanics. Specifically, static single-leg radiographs do not directly measure gait asymmetry, knee adduction moment, or varus thrust. The absence of gait analysis and objective biomechanical assessment is therefore an important limitation. Prospective studies combining radiographic assessment with gait analysis, quantitative loading measurements, and clinical outcomes are warranted to determine whether the observed radiographic changes have functional or therapeutic significance.

## 5. Conclusions

Although numerically modest, the observed increase in varus angle suggests that single-leg weight-bearing radiographs may reveal load-dependent differences in coronal alignment that are not fully captured by standard bilateral weight-bearing radiographs. However, whether incorporating single-leg weight-bearing radiographs into preoperative assessment and surgical planning, particularly for alignment-focused procedures such as HTO and TKA, provides clinically meaningful additional information remains to be established. Future prospective studies integrating single-leg weight-bearing radiographs with gait analysis, objective biomechanical assessments, and clinical outcomes are needed to clarify the relationship between load-dependent radiographic alignment and functional knee biomechanics and to determine whether these additional measurements improve surgical decision-making.

## Figures and Tables

**Table 1:** Comparison of demographic characteristics and radiographic measurements according to sex.

	Female (*n* = 57)	Male (*n* = 44)	*p*
**Age**	58.9 ± 11.9	54.5 ± 13.4	0.080
**Dominant Knee**			0.757
**Right**	43	32	
**Left**	14	12	
**BMI**	24.8 ± 3.1	25.5 ± 2.1	0.201
**Right Knee ΔVarus**	1.59 ± 0.82	1.47 ± 0.66	0.375
**Left Knee ΔVarus**	1.28 ± 0.75	1.34 ± 0.64	0.706
**Right mMPTA**	86.7 ± 2.8	86.7 ± 3.1	0.934
**Left mMPTA**	86.4 ± 1.8	86.7 ± 1.5	0.288
**Right mLDFA**	89.5 ± 2.9	89.3 ± 2.5	0.394
**Left mLDFA**	89.2 ± 1.2	89.2 ± 0.97	0.703
**JLCA**	3.2 ± 1.2	3.3 ± 1.5	0.907
**MAD**	4.0 ± 1.2	4.3 ± 1.4	0.375
**PTS**	6.9 ± 1.5	6.7 ± 1.8	0.418

**Table 2:** Comparison of varus angle change after single-leg weight bearing.

		95% Confidence Interval		
	ΔVarus (°), Mean ± SD	Lower	Upper	*p*
**Right Knee ΔVarus of Full Cohort**	1.54 (0.75)	1.39	1.69	<0.001
**Left Knee ΔVarus of Full Cohort**	1.30 (0.70)	1.16	1.44	<0.001
**Right Knee ΔVarus in right-dominant patients**	1.68 (0.71)	1.51	1.84	<0.001
**Left Knee ΔVarus in left-dominant patients**	1.57 (0.80)	1.25	1.90	<0.001

**Table 3:** Correlation of radiographic parameters with ΔVarus in both knees.

	Right Knee ΔVarus					Left Knee ΔVarus			
	Spearman’s ρ	95% Confidence Interval		*p*		Spearman’s ρ	95% Confidence Interval		*p*
		Lower	Upper				Lower	Upper	
**Right m MPTA**	−0.001	−0.190	0.197	0.993	**Left m MPTA**	0.085	−0.125	0.289	0.399
**Right m LDFA**	−0.058	−0.244	0.144	0.567	**Left m LDFA**	−0.097	−0.297	0.113	0.336
**JLCA**	0.156	−0.047	0.349	0.120	**JLCA**	−0.100	−0.282	0.078	0.319
**MAD**	0.078	−0.121	0.294	0.439	**MAD**	−0.129	−0.327	0.072	0.199
**PTS**	−0.199	−0.392	0.009	0.046	**PTS**	−0.045	−0.227	0.134	0.658

**Table 4:** Multivariable linear regression analysis of factors associated with right and left knee ΔVarus.

		Right Knee ΔVarus		
	B	95% Confidence Interval	Standardized β	*p*
**Age**	0.006	−0.006 to 0.019	0.107	0.302
**BMI**	0.038	−0.017 to 0.094	0.141	0.174
**Right mMPTA**	0.015	−0.037 to 0.067	0.059	0.575
**Right mLDFA**	−0.029	−0.086 to 0.027	−0.108	0.305
**JLCA**	0.086	−0.027 to 0.199	0.156	0.136
**MAD**	−0.007	−0.121 to 0.106	−0.013	0.899
**PTS**	−0.064	−0.154 to 0.025	−0.146	0.157
		**Left Knee ΔVarus**		
**Age**	−0.011	−0.022 to 0.000	−0.199	0.060
**BMI**	−0.006	−0.058 to 0.046	−0.023	0.828
**Left mMPTA**	0.033	−0.050 to 0.117	0.081	0.428
**Left mLDFA**	−0.028	−0.155 to 0.100	−0.045	0.669
**JLCA**	−0.061	−0.169 to 0.047	−0.119	0.263
**MAD**	−0.039	−0.149 to 0.072	−0.073	0.488
**PTS**	−0.045	−0.129 to 0.040	−0.109	0.295

## Data Availability

Data are available upon reasonable request.

## Abbreviations

The following abbreviations are used in this manuscript:

HTO	High tibial osteotomy
TKA	Total knee arthroplasty
OA	Osteoarthritis
mMPTA	Mechanical Medial Proximal Tibial Angle
mLDFA	Mechanical Lateral Distal Femoral Angle
JLCA	Joint Line Convergence Angle
PTS	Posterior Tibial Slope
MAD	Mechanical axis deviation
